# Immunopathophysiology of Juvenile Spondyloarthritis (jSpA): The “Out of the Box” View on Epigenetics, Neuroendocrine Pathways and Role of the Macrophage Migration Inhibitory Factor (MIF)

**DOI:** 10.3389/fmed.2021.700982

**Published:** 2021-10-06

**Authors:** Miroslav Harjacek

**Affiliations:** Department of Pediatrics, College of Medicine and Health Sciences, United Arab Emirates University, Al Ain, United Arab Emirates

**Keywords:** juvenile spondyloarthritis, enthesitis-related arthritis, pathophysiology, epigenetics, neuroendocrine pathways, hypothalamic-pituitary axis, gut-joint axis, macrophage migration inhibitory factor

## Abstract

Juvenile spondyloarthritis (jSpA) is a an umbrella term for heterogeneous group of related seronegative inflammatory disorders sharing common symptoms. Although it mainly affects children and adolescents, it often remains active during adulthood. Genetic and environmental factors are involved in its occurrence, although the exact underlying immunopathophysiology remains incompletely elucidated. Accumulated evidence suggests that, in affected patients, subclinical gut inflammation caused by intestinal dysbiosis, is pivotal to the future development of synovial–entheseal complex inflammation. While the predominant role of IL17/23 axis, TNF-α, and IL-7 in the pathophysiology of SpA, including jSpA, is firmly established, the role of the cytokine macrophage migration inhibitory factor (MIF) is generally overlooked. The purpose of this review is to discuss and emphasize the role of epigenetics, neuroendocrine pathways and the hypothalamic-pituitary (HPA) axis, and to propose a novel hypothesis of the role of decreased NLRP3 gene expression and possibly MIF in the early phases of jSpA development. The decreased NLRP3 gene expression in the latter, due to hypomethylation of promotor site, is (one of) the cause for inflammasome malfunction leading to gut dysbiosis observed in patients with early jSpA. In addition, we highlight the role of MIF in the complex innate, adaptive cellular and main effector cytokine network, Finally, since treatment of advanced bone pathology in SpA remains an unmet clinical need, I suggest possible new drug targets with the aim to ultimately improve treatment efficacy and long-term outcome of jSpA patients.

## Literature Search

A systematic literature search was conducted in Ovid Medline (PubMed), Scopus, Science Citation Index expanded, and Google Scholar to find related articles. The key words were “juvenile spondyloarthirtis”, “enthesitis-related arthritis (ERA)”, “pathophysiology”, “signal transduction”, “epigenetics”, “neuroendocrine pathways”, “stress response”, “HPA axis”, “sex hormones”, “gene expression”, “proteomics”, “Gut–joint axis”, “dysbiosis” “NLRP3”, “tissue hypoxia”, “new bone formation (NBF)” in combination with “macrophage migration inhibitory factor (MIF)”. I also manually screened the reference lists in relevant reviews and other non-primary data sources captured by the search strategy. Only publications in English were included.

## Introduction

Spondyloarthritis (SpA) is an umbrella term for a group of chronic inflammatory disorders that share common clinical and pathophysiological features. In children, enthesitis-related arthritis (ErA) is a subgroup of juvenile idiopathic arthritis (JIA) clinically characterized by enthesitis, chronic inflammatory arthritis, acute anterior uveitis, back pain, and low-grade gut inflammation. ErA also falls under the collective term of juvenile spondyloarthritis (jSpA) (1). Depending on the geographic region, ErA accounts for 15–30% JIA cases and is one of the commonest subtype of JIA seen in Asia (2). The jSpA commonly starts as “undifferentiated” disease (e.g., ERA) which differs between children and adults. For example, in juvenile-onset disease (jSpA), when compared to adults, hip arthritis is more frequently observed, there is a lower prevalence of human leukocyte antigen B27 positivity, axial involvement and acute anterior uveitis, but less peripheral arthritis and enthesitis (1). Although several classification criteria are used in children for uniformity of diagnoses, several other conditions share similar clinical features, thus resulting in either overlap or indistinct classifications. The juvenile spondyloarthritis (JSpA) is a perfect example of that, as the currently available criteria do not reflect their complexity and peculiarities (3).

The SpA family of diseases comprises undifferentiated jSpA (ERA), ankylosing spondylitis (AS), reactive arthritis (ReA), psoriatic arthritis (PsA), and inflammatory bowel disease (IBD) associated arthritis. In general, SpAs are depicted by inflammation, bone erosions and new bone formation (NBF). Enthesis, representing the connective tissue junction where ligaments and tendons attach to the bone, is a primary target tissue for inflammation in SpA, with inflammation affecting both the enthesis soft tissue and the nearby anchoring peri-entheseal bone (PEB) (4). In fact, the anchoring PEB, or synovial–entheseal complex, is the main site of inflammation and osteitis in SpA (5).

As expected, the majority of the published genetic studies in SpA have been restricted to ankylosing spondylitis (AS), the classical form of SpA in adults. The genetic heritability of juvenile spondyloarthropathy remains incompletely understood, with HLA-B27 accounting for almost 25% of its identified heritability, with newly discovered gene mutations responsible for 2.1% of inherited cases (6). However, studies have shown that HLA-B27 positivity on its own is not sufficient to trigger disease as the concordance rate for HLA-B27 positivity in dizygotic twins was shown to be significantly lower than for monozygotic twins (24 vs. 63%, respectively), suggesting the important role of other relevant genes with an oligogenic model of familial transmission (7).

## Gene Expression and Proteomic Studies

Recent genome-wide association studies (GWAS) and single nucleotide polymorphisms (SNPs) analysis have further delineated the role of non-MHC genes in the development of adult AS, involving the interaction of endoplasmic reticulum aminopeptidase 1 (ERAP1) with HLA-B27 (8). The role of ERAP1 was also later confirmed for ErA and IL-23R for juvenile psoriatic arthritis (9). ERAP1 polymorphisms only affect the risk of development of SpA in HLA-B27-positive individuals, suggesting that they influence SpA pathogenesis by altering HLA-B27 function (10). Nevertheless, for better understanding of differences between genotype and phenotype as well as mechanisms of disease development, research methods such as quantification of gene expression are often necessary. So far, although a number of different gene expression studies in adult patients have been conducted, they included only a small number of patients with jSpA (11–13). In another cohort of patients diagnosed with ErA, using ILAR criteria, and with a known HLA genotype, none of the transcriptome studies was performed with RNA isolated from whole blood, nor was the calculation of the odds ratio (OR) for disease development performed, with absence of independent verification of data specificity and universality. Our group conducted a meticulous gene expression analysis in a very homogenous group of Croatian patients with enthesitis-related arthritis (ErA) diagnosed according to ILAR classification criteria. We documented increased expression of TLR4 and CXCR4 and decreased expression of NLRP3 and PTPN12 genes (13). In another ErA cohort from the USA, Barnes et al., found different genes or gene clusters, resulting in the under-expression of hemoglobin genes, with unknown significance so far (11). In a different study by Myles et al. involving Indian patients with ErA, gene expression in synovial fluid mononuclear cells (SFMCs) was compared to that in peripheral blood mononuclear cell (PMBCs). SFMCs were found to have a different gene expression profile from PBMCs, with overexpression of genes associated with various cell processes such as antigen presentation, scavenger function, chemotaxis and proteases, while genes involved in NK cell function, cell adhesion and inhibitors of apoptosis were under-expressed, suggesting a dysregulation of the innate immune system genes in that condition (12). The mechanism(s) responsible for those alterations, which differ among populations, remain largely unknown (see below).

Gene expression studies provide important information about the involvement of various signal pathways. They rely, however, on plasma which is frequently used as surrogate, instead of the synovial membrane proteome, actual site of pathology, from early disease-stage, which would be most informative for precise determination of immunopathology of jSpA. However, synovial membrane is extremely difficult to acquire. Although control tissue from healthy children would have been useful, ethical considerations prevent it. On the other hand, as synovial fluid (SF) is in close proximity to tissues primarily altered during jSpA, analyzing it has significant potential to better understand the underlying immunopathogeneses. In the study of Rozenkranz et al. distinctively 24 proteins were identified as differentially abundant in SF between JIA subtypes, but jSpA patients were not included (drugi link). However, in the Taiwanese pilot study of two children with diagnosed enthesitis-related arthritis (ERA), the patients' plasma was studied before and after the administration of etanercept alone, using conventional two-dimensional gel electrophoresis (2-DE) in combination with mass spectrometry (MALDI-MS). They showed that etanercept therapy improved clinical ERA symptoms through the regulation of several cytokines (IL-2/IFN-γ), chemokines (MCP-1), and growth factors (GRO) that affect the expression of specific acute phase proteins such as haptoglobins, immunoglobulin A, and fibrinogen-γ chain (14). However, there are many challenges within the SF proteomics field including the requirement for standardized and stringent methods of sample collection and storage, the differences in sensitivity and specificity of various proteomic assays, the impossibility of including healthy controls, compounded by the lack of comprehensive biostatistical analysis of the data to exclude falsely detected biomarkers (15).

## Epigenetic Studies

The role of epigenetic mechanisms is essential in the regulation of gene expression, and consequently in the pathogenesis of various diseases, including rheumatic conditions (16, 17). The notion that these mechanisms could be influenced by external stimuli raises the possibility of a link between the environment and gene function, providing a potential clue for the potential contribution of these external stimuli to many diseases. Epigenetic mechanisms are traditionally defined as mitotically and/or meiotically heritable changes in gene expression that do not involve changes in DNA sequence. To contribute to the control of gene expression and repression, they are closely connected with other regulatory elements, such are transcription factors, and in some cases, with extracellular factors such as cytokines and growth factors (17). Although various studies have already confirmed the prevalence of epigenetic changes in both genetically complex and monogenic inflammatory rheumatic diseases, such are rheumatoid arthritis (RA), systemic lupus erythematosus (SLE), systemic sclerosis (SSc), Sjorgen syndrome (SS), Cryopyrin-associated periodic syndrome (CAPS) and Familial Mediterranean Fever (FMF), to best of our knowledge, none has looked at patients with juvenile spodyloarthritis (16–19). In oligo-JIA, Chavez-Valencia et al. have found no substantial alterations in DNA methylation of CD4^+^ T cells, but only modest alterations in genes of known or potential relevance to JIA (20). On the other hand, DNA methylation of the pro-inflammatory interleukin-32 (IL-32) gene was found to be reduced in JIA CD4+ T cells, suggesting an association between the reduction of IL-32 methylation and JIA (21). At present, there are at least three accepted mechanisms that can initiate and maintain epigenetic alterations: DNA methylation as pretranscriptional, histone modifications and non-coding RNA (ncRNA)-associated gene silencing like microRNAs (miRs) at the posttranscriptional level (22, 23). In our recent study in patients with ErA, we assessed the methylation levels of the *TLR4, CXCR4, NLRP3*, and *PTPN12* gene promoter, as well as the expression of several non-coding microRNAs (miR-150, miR-146a, miR-181a and miR-223) with reported interactions with the specific genes we were interested in. We collected PBMCs from 19 newly diagnosed patients with jSpA, according to ILAR classification criteria for enthesitis-related arthritis (ErA), and seven gender- and age-matched asymptomatic children. Out of four genes studied, we only found hypermethylated NLRP3 gene, while the expression analysis of selected microRNAs showed no significant difference (24, 25). DNA methylation studies in adults with AS have already identified over 1600 hypermethylated loci in the peripheral blood, most of which are located in HLA genes (26). In other studies, genes such as DNMT1 and BCL11B were found to be hypermethylated, but their expression did not correlate with the clinical manifestations of ankylosing spondylitis (27, 28). In a similar study of patients with AS, Coit et al. demonstrated an overexpression of hypermethylated genes like GTPase-related genes, as well as hypomethylated genes that included HCP5 gene encoding a lncRNA within the MHC region linked with a genetic risk for psoriasis and toxic epidermal necrolysis. Furthermore, the presence of an HLA-B*27 allele was associated with strong hypomethylation of HCP5, tubulin folding cofactor A (TBCA) and phospholipase D Family Member 6 (PLD6), of unknown relevance at this point (29). On the other hand, the miRNA expression profiles in the blood of patients with AS showed 19 differentially expressed miRNAs, with increased levels of miR-146a and miR-155 compared to controls, and with the disease index correlating only with miR-155 expression (30). Furthermore, IL-10 inhibits the pro-inflammatory microRNA miR-155 through STAT3 (31). This is relevant in the context of SpA where generally low IL-10 values are found in patients across the different phenotypes (*see below*).

## Stressors Exposure and Neuroendocrine Immuno-Modulation

When environmental strains exceed the human adaptive capacity or ability to cope, stress will occur. These environmental strains are collectively termed stressors, and appropriate responsiveness of the stress system to stressors translates into a sense of general wellbeing, adequate task performance and positive social interactions (e.g., homeostasis) (32, 33). By contrast, the effect of various stressors may hamper a child's growth and development, and may be responsible for various rheumatology, endocrine, metabolic, immune-mediated and psychiatric disorders (33). For example, in the extensive Swedish cohort of 2,453 adults with AS, increased risk for disease development was linked to respiratory tract infections in childhood (34). As shown in both adult and pediatric patients with rheumatological conditions, the occurrence of stressful or traumatic life events frequently precede the onset of their illness or disease flares. It is well known that trauma or mechanical stress are frequent triggers or flare-inducers of JIA, and particularly for the induction of enthesitis (35). Moreover, stress can cause the brain to trigger the immune response, which can, in turn, induce changes in the central nervous system (CNS) suggesting bidirectional communication. However, in the course of chronic inflammation, an interruption of this communication might be possible.

Based on the observation of sex differences in AS, in studies performed over 50 years ago an etiological association with endocrine factors was suggested. A study of testicular function in 22 patients with AS demonstrated diminished testicular testosterone (T) reserve, elevated luteinizing hormone (LH) serum level, estradiol/testosterone ratio (E2: T) inversion and slightly increased estradiol (E2) serum level (36, 37). Interestingly, in the animal SKG mouse model *(SpA model*), estrogen was found to suppress TNF-α and arthritis development (38). Similarly, other animal model studies have shown that estrogen can suppress the differentiation of T helper (Th)17 cells from naive T cells (39).

Low serum levels of sex hormones, especially dehydroepiandrosterone sulfate (DHEAS) (i.e., **androgen drain**), may also contribute to bone loss in patients with AS, while patients with early or adult reactive arthritis have a high cortisol and DHEA serum levels that might change the course of disease (40, 41). After administration of a low-dose of adrenocorticotropic hormone (ACTH), the serum cortisol rise became significantly lower in patients with AS than in controls, suggesting an impaired hypothalamic-pituitary-adrenal (HPA) axis and reinforcing the possibility of involvement of the neuroendocrine system in the etiology of AS (“**the disproportion principle**”) (42). More importantly, low cortisol and testosterone serum levels were found in subjects with active JIA, while the lowest androgen levels were found in those patients in whom disease extended into their adult life (43). In addition, Bravo et al. found elevated levels of serum prolactin (PRL), another pro-inflammatory hormone, in male patients with juvenile ankylosing spondylitis, with levels correlating with disease activity (44). The detailed description of the neuroendocrine pathways including HPA, arousal/sympathetic nervous system (SNS) and parasympathetic nervous system (PNS), are beyond the scope of this paper but have been detailed in other reviews (45–51).

**Figure 1:**
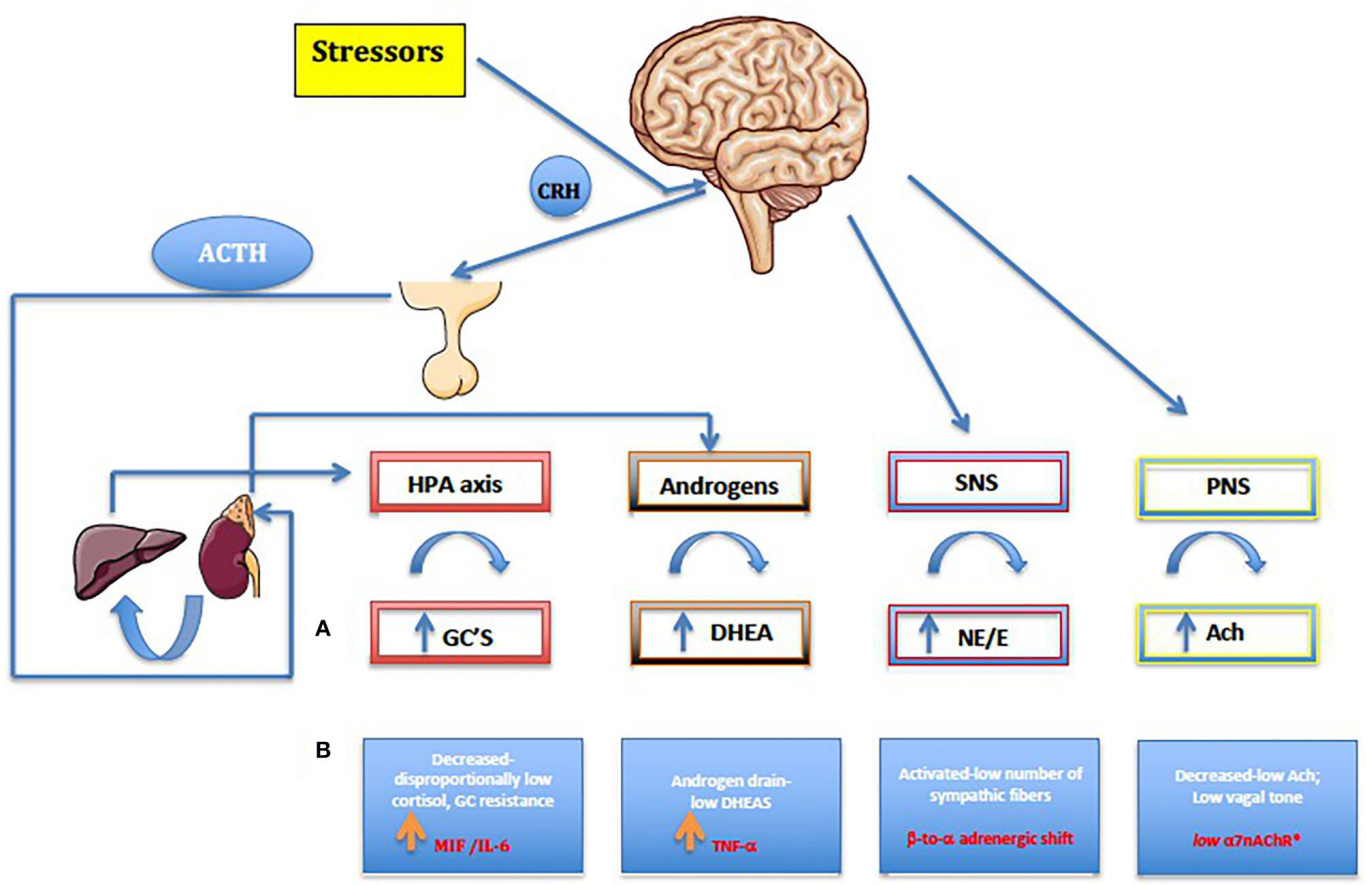
**(A)** Normal, healthy neuroendocrine pathways seen also in reactive and early undifferentiated arthritis. **(B)** Chronic arthritis including jSpA. Along with the autonomic nervous system (SNS and PNS), the HPA axis is the main stress response mechanism, forming a carefully regulated signaling network. HPA axis dysregulation results in various downstream physiological consequences, like increasing risk for immune-mediated disease, mood disorders, metabolic disease, and cardiovascular disease. On the other hand, gonadal hormones play a master role in the formation, activation, and regulation of HPA axis. By affecting the response and sensitivity to cytokines, neurotransmitters, and hormones, gonadal steroids help to HPA axis to fine-tune the levels of stress hormones in the circulation. Furthermore, the behavioral responses to stress and obvious sex differences are particularly important considering their role in maintaining homeostasis. Pro-inflammatory cytokines namely MIF, TNF-α and IL-6 have a major role in shaping up final anti-stressor response during the chronic inflammation. Furthermore, centrally produced MIF by pituitary gland, was the first cytokine that can counter-regulate the inhibitory effects of glucocorticoids and, as a result, plays a critical role in the host control of inflammation and immunity in general. Imbalance in the autonomic nervous system (ANS) or dysautonomia has been detected in many established chronic autoimmune diseases, and in general, patients with chronic synovitis have increased activation of sympathetic fibers *(ß* to α adrenergic shift) and reduced PNS activity (*low a7nAChR*). For the details see text (32–65). HPA, hypothalamic-pituitary-adrenal axis; CRH, corticotropin-releasing hormone; ACTH, adrenocorticotropic hormone; GC, Glucocorticoids; ACh, acetylcholine; NE, norepinephrine; E, epinephrine; DHEA, dehydroepiandrosterone.

The role of the HPA axis extends to the glucocorticoid (GC) metabolism. Cortisol is converted to cortisone mainly by the kidney, *via* 11β-hydroxysteroid dehydrogenase (11β-HSD) type 2, while the major organ for converting cortisone back to cortisol is the liver, *via* 11β-HSD1. Interestingly, in arthritis, conversion from cortisone to cortisol by 11β-HSD1 is increased (48). In addition, the circadian rhythm of the HPA axis may be defective in overcoming the signs and symptoms of the disease associated with inadequate cortisol secretion. This may augment negative feedback and explain the HPA dysfunction in inflammatory conditions (49). On the other hand, macrophage migration inhibitory factor (MIF) is secreted from identical corticotrophic pituitary cell type know to secrete ACTH, the hormone that stimulates the adrenal secretion of glucocorticoids. MIF was shown *in vivo* to neutralize the glucocorticoid-induced suppression of inflammatory cytokine secretion in activated macrophages (i.e. TNF-α, IL-1-β, IL-6, IL-8) (Figure 1) (52). The circadian variation in plasma MIF closely parallels glucocorticoid levels. During stressful events or life-threatening infections coupled with high levels of glucocorticoids, the antagonistic effects of MIF on glucocorticoids probably represent the mechanism by which the host preserves a functioning immune response (52). Ralph et al. provided evidence that described the nuclear orphan receptor 1 (NURR1) as a target of MIF and GCs in RA, and a repressor of MAPK phosphatase 1(MKP1) expression. MKP1, the negative regulator of MAPK activation has been identified as a key gene that regulates MIF. Thus, NURR1, target of both GCs and MIF in mediating their opposing effects on MKP1, appears to be involved in the vital regulatory network that influences both innate and adaptive immune responses, as well as disease phenotypes (53). There is therefore a clear transition from a well-functioning HPA axis in the early phases of undifferentiated spondyloarthritis or reactive arthritis, to an inhibited HPA axis in late stages of chronic spondyloarthritis. This phenomenon is thought to be due to pro-inflammatory cytokine production such as MIF, IL-6, TNF-α and IFN-γ, which are likely to negatively influence steroidogenesis. Thus, in chronic spondyloarthritis, MIF might counter-regulate the suppressive effect of glucocorticoids on cytokine mRNA translation (Figure 1).

The combination of reduced parasympathetic with increased sympathetic tone, has been a consistent finding in chronic adult arthritis patients, suggesting an imbalanced autonomic nervous system (53–56). The sympathetic nervous system (SNS) has a bimodal effect in the chronic arthritis, by either increasing or decreasing serum levels of proinflammatory and anti-inflammatory cytokines. This depends on several factors, such as the time point of immune system activation, the cellular context, and the distinct adrenoceptors involved (α vs. β) (54). Although there is no published evidence in juvenile spondyloarthritis, the peripheral blood mononuclear cells (PBMC) of adult patients with JIA express mRNA-encoding α-adrenergic 1 receptors (α1-AR subtype), which are not found in healthy children. It seems, therefore, that the expression of α1-AR mRNA in PBMC during chronic inflammation might be associated with attenuated immune responses to stress (57). Functional α1-AR receptors seem to be upregulated on the leukocytes of patients with poliJIA, resulting in higher IL-6 levels upon stimulation of these receptors by a cold pressor test (58). Consequently, the α-ARs might become more relevant in a later stage of chronic inflammation, concurring with decreased numbers of β-ARs (“**β-to-α-adrenergic shift”)** (59). The endogenous synergy of HPA axis (cortisol) and SNS is clearly demonstrated in patients with chronic synovitis, by the stiffness and/or the decrease of high cytokine levels in the morning (47).

On the other hand, the immunosuppressive effect of the parasympathetic nervous system is much more obvious. The cholinergic anti-inflammatory pathway (e.g., “**anti-inflammatory reflex”**) can suppress inflammation through release of acetylcholine (ACh) by the vagus nerve, involving the alpha7 nicotinic acetylcholine receptor (alpha7nAChR) expressed on CD68+ macrophages and other immune cells (60–62). As a result, a series of well-known proinflammatory molecules such as TNF-α, IL-6, MIF, IFN-γ, high-mobility group box-1 (HMGB-1), free radicals, inducible nitric oxide (iNO), and others are inhibited (61). Interestingly, both the α7nAChR, ubiquitously expressed by CD4^+^T lymphocytes, and the nAChR agonist nicotine, can inhibit the production of IL-17 in CD4^+^ T cells in human peripheral blood (63). In various animal models, such as those for arthritis and colitis, nicotine has also been shown to have an anti-inflammatory effect by inhibiting the polarization to Th1/Th17 (64). This mechanism has been reinforced in a small pilot study of 37 adult patients with psoriatic arthritis or AS, where a transcutaneous vagal nerve stimulation (t-VNS) led to a significant reduction in ASAS scores (65).

## Subclinical Gut Inflammation (Gut–Joint Axis)

The gut epithelial barrier is a first line of defense against harmful microorganisms. Disruption of the epithelial layer puts gut microbes in direct contact with the host's immune cells, thereby activating an aberrant inflammatory response. It has been shown that prenatal and early life bacterial gut colonization is thought to play a paramount role in shaping the immune system. This is translated into the gain of basic functions such as immunotolerance of commensal microorganisms. Early life exposures have been linked to the development of inflammatory bowel disease (IBD) later in life. Infants born to mothers with IBD demonstrated enrichment in *Gammaproteobacteria* in their gut, often associated with intestinal inflammation, and also depletion in protective *Bifidobacteria*. Likewise, germ-free mice (GFM) inoculated with stools of third trimester IBD mother and of 90-day infants, showed a significant reduction in microbial diversity and fewer class-switched memory B cells and regulatory T cells in the colon (66). A study by Yatsuneko et al. suggests that gut microflora evolves toward a stable configuration by the age of 3 years (67). On the other hand, subclinical gut inflammation is a hallmark in all forms of juvenile spondyloarthritis and is associated with a high prevalence of inflammatory bowel disease (IBD) (66). The involvement of the gut–joint axis of inflammation in jSpA is strengthened by similarities in immunopathogenesis, and also by the clinical success of anti- TNF-α and IL-23 therapies in both IBD and in some forms of SpA (67). It is believed that inflammation in SpA originates in the gut and subsequently leads to joint inflammation. Both conditions share many genetic risk factors as well as changes in the composition of gut microbiota. Although conceptually attractive, some therapies targeting IL-17A are efficacious in the joint but not *vice versa*, and the targeting of adhesion molecules such as α4β7 in IBD can lead to onset or flares of SpA (67). Recent studies in ethnically different patient populations, and especially in patients with HLA-B27, have demonstrated dysbiosis in patients with SpA (68–76). Such dysbiosis is highly dependent on the host's genetic background and/or environment, implicating an “**ecological model of dysbiosis”**, with the effects of a multitude of microbes contributing all to the aberrant immunopathogenesis (77). At the functional level, different inflammation-associated microbes exhibit common metabolic pathways, including the synthesis of short-chain fatty acids (SCFA) such as butyrate, steroid biosynthesis as well as bacterial motility (78). The synthesis of butyrate, which has anti-inflammatory effects, promotes the development of regulatory T cells and is generally decreased in patients with SpA (78). The metabolomics data, in addition to less convincing 16S data, suggest differences in tryptophan metabolism in children with ErA, linked to the fecal microbiota, with a pro-inflammatory effect (79). Furthermore, as shown in early AS and HLA-B27 positive adults, dysbiosis and a leaky gut lead to adaptive immune activation associated with characteristic MRI phenotype of osteitis (80). Whether these changes are intrinsically inherent to the disease, or are a mere consequence of a more systemic inflammatory process that also involves the intestine it is not clear at this point. However, data from animal models and studies on relatives of patients with SpA, strongly suggest that these changes indeed precede the onset of the disease (76). In a some rheumatic disease it is possible that the use of specific probiotics, as an adjuvant therapy, correct the dysbiosis, resulting in the overall clinical efficacy. Our preliminary data has shown that VSL-3 medical probiotic, with a proven role in IBD, can improve clinical symptoms and decrease disease activity in jSpA patients (81). On contrary, different proof-of-concept studies from India, showed no clinical or immunological benefit in patients with JIA-ERA, with VSL-3 probiotic use compared to the regular use of NSAIDs (82).

## The Role of Macrophage Migration Inhibitory Factor (MIF)

While the dominant role of IL17/23 axis, TNF-α, and IL-7 in pathophysiology of SpA, including jSpA, is well established, the role of cytokine MIF has generally been overlooked (83–89). The MIF is a critical upstream alarming-like mediator of innate immunity and inflammation. Under physiological conditions MIF circulates with serum concentrations between 2 and 6 ng/ml, with a circadian rhythm correlating with plasma cortisol (90). As mentioned earlier, it plays a pivotal role in the neuroendocrine axis mediated tissue-specific damage mechanisms, by counteracting the immunoregulatory effects of glucocorticoids (GCs) (52, 90). Unlike other cytokines, MIF is intrinsically expressed and stored in intracellular granules of various immune cells such as T- and B- lymphocytes, monocytes, macrophages, dendritic cells (DCs), mast cells, neutrophils, basophils, endothelial cells, tissue macrophages, and certain parenchymal cells (91). In response to liposaccharides (LPS) and stress, MIF is released from preformed cytoplasmic pools of mainly macrophages and dendritic cells. It up-regulates the expression of pattern recognition receptors, induces synthesis of downstream inflammatory cytokines, including IL-1β, IL-6, TNF-α, IFN-γ, IL-17 and sustains the inflammatory responses by inducing recruitment of neutrophils, monocytes, macrophages and DCs and inhibiting their activation-induced apoptosis (92, 93). In order to regulate autophagy/mitophagy as well as glucose catabolism, MIF induces, in an autocrine or paracrine manner, enhancement of phagocytosis and an increase of the production of reactive oxygen species (ROS) and nitric oxide (NO) (94–96). In humans, MIF, a 114-amino-acid non-glycosylated peptide of 12.5 kDa, is encoded by a single gene located on chromosome 22q11.2m (92). Two distinct polymorphisms of MIF exist: rs755622 (– 173 G > C) and rs5844572 (– 794 CATT tandem repeat). They exist in linkage disequilibrium, and are associated, in different proportions, with various autoimmune diseases, such as SLE, systemic onset JIA, psoriasis and ulcerative colitis (97–100). Depending on the cellular context and disease state, MIF signaling is mediated by its receptors CXCR2, CXCR4 and/or CD74. The latter receptor alone mediates extracellular MIF binding, but MIF-induced MAPK signaling requires the co-expression of hyaluronan receptor CD44 leading to subsequent activation of proinflammatory transcription factor nuclear factor- κβ (NF-κβ) (101). The noncognate binding of MIF to CXCR2 and CXCR4 is the molecular basis for MIF-triggered recruitment of monocytes and T cells (102). In T cells and fibroblasts activation of JNK signaling by MIF involves the upstream kinases PI3K and SRC and is dependent on CXCR4 and CD74 (101). Besides, MIF inhibits p53-mediated apoptosis in macrophage with the induction of increased cytoplasmic phospholipase A2 (PLA2), arachidonic acid, COX2 and PGE2, which maintains the macrophage pro-inflammatory function (102). Increased gene expression of CD74 occurs in inflamed and noninflamed colonic mucosa of IBD patients, and it is also a possible T cell antigen in SpA, eliciting Th1 and Th17 responses (103, 104).

Intracellular MIF is involved in Toll-like receptor and inflammasome-mediated inflammatory responses. It upregulates Toll-like receptor 4 (TLR-4) expression, and consequently induces the release of proinflammatory cytokines such as TNF-α and interleukin IL-12, known to play an important role in pathogenesis of SpA (105). Loss of MIF has been shown to suppress the LPS-induced release of TNF-α by downregulating TLR4 expression (105). In response to the stimulation by LPS and Gram-negative bacteria (*canonical TLR4 activators*), the MIF-deficient macrophages have reduced production of TNF-α and IL-6, underlining a role for MIF in modulation of TLR4 downstream signaling pathways (106). We have already proposed that the Thr399Ile polymorphism of TLR4, found in variant carriers of Croatian patients with jSpA but undetectable in Indian patients, may be accountable for modified immune response to microbial infection (107, 108). MIF, via an interaction with JAB1/ CSN5, directly affects transcriptional activity of activator protein-1 (AP-1), a central regulator of several proinflammatory genes (109). This hints to a possibly interesting overlap between MIF and glucocorticoid mediated (GC) responses. An important mechanism of GC action is the ability to suppress AP-1- and NF-κβ-regulated genes, with steroid-resistant disease being often associated with increased AP-1 activity (110). Moreover, MIF is either directly involved in the assembly and activation of the NLRP3 inflammasome, or via intermediate filament protein vimentin, which is essential for NLRP3 activation (111). More importantly, this role is independent of its function as a cytokine, because recombinant and native MIF are unable to salvage NLRP3-dependent IL-1 release in *Mif*^−/–^ macrophages (111). Depletion or inhibition of MIF in macrophages and DCs result in the inhibition of IL-1α, IL-1β and IL-18 in response to NLRP3-activating stimuli. It appears, therefore, that by regulating NLRP3 inflammasome activation and downstream IL-1β production, MIF has an upstream role in outlining the inflammatory characteristics of activated macrophages and DCs (111). Activation of caspase-1 is the main characteristic of inflammasome activation, with higher caspase-1 serum level in SpA, gout, inflammatory arthritis, and osteoarthritis than in other conditions (112).

Moreover, hypoxia initiated by microbiotome, plays a physiologic role in the normal intestine, and has also a disease-perpetuating role in the intestines of IBD patients (113). The oxygen used for butyrate metabolism is an important factor of intestinal homeostasis. Butyrate has a dual role: it is the primary fuel source for the colon, and also shapes the gut microbiotome (114). Hypoxia stabilizes hypoxia-inducible factor (HIF), a transcription factor that regulates many genes important for intestinal barrier function (115, 116). In addition, following a hypoxic stimulus, innate immune cells, including neutrophils, macrophages and dendritic cells, resist apoptosis, and in addition, intra-epithelial cells (IECs) are stimulated to produce TNF-α and other pro-inflammatory cytokines, causing increased barrier permeability (e.g., leaky gut) (117). This effect is furthermore perpetuated by oxygen consumption by the luminal bacteria, and also by inflammatory mediators and LPS, which also regulate HIF activity (117). The chronic HIF stimulation in the colon epithelial cells initiates a hyperinflammatory reaction and, at least in mice, HIF-1α enhances experimental colitis through a MIF-dependent inflammatory signaling cascade, reversed by MIF inhibition (118). This autoamplifying feedback loop could be interrupted by high doses of GCs via the GCR, or by the inhibition of HIF-1 an expression/stabilization under normoxia (119). MIF-JAB1 interaction also stabilizes HIF1α by preventing its hydroxylation, resulting in increased expression of pro-angiogenic factor such as VEGF (120, 121). These observations support the view that hypoxia is a key driving factor in chronic inflammation, and in case of jSpA on both gut and joint levels. Hypoxia, and in particular HIF-1α, are very potent inducers of MIF in the joints, as shown in cultured RA synovial fibroblasts stimulated by rhMIF (121). In macrophage cultures, hypoxia induces TLR-4 which is also important in the context of jSpA (122). Therefore, accumulating evidence supports hypoxia and HIFs in regulating a number of important pathophysiological characteristics of chronic arthritis, including synovial inflammation, angiogenesis, and cartilage destruction (123).

## Suggested Novel Hypothesis

Following the above discussion, I suggest a novel hypothesis in which decreased NLRP3 gene expression, due to epigenetic modifications of promotor site, is (one of) the cause for inflammasome malfunction leading to gut microbiota composition alterations observed in patients with early jSpA. This dysbiosis (*caused by NLRP3 dysfunction*) could potentially cause increased influx of TLR4 ligands and increased expression of the TLR4 gene (*possibly due to Thr399Ileu polymorphism of TLR4*), reduction of commensal bacteria with anti-inflammatory properties, namely *Faecalibacterium prausnitzii*, known to inhibit NF-κβ signaling, and finally leading to TNF-α abundancy, characteristic of jSpA (124).

The reduced expression of NLRP3 gene is a new and intriguing observation in jSpA/ErA. Studies on the role of NLRP3 inflammasome in IBD yielded controversial results. Earlier studies have reported that activated NLRP3 inflammasome stimulated production of IL-1β and IL-18 and contributed to intestinal inflammation (125). However, the concept of damaging inflammasome signaling in IBD is currently being reconsidered. This follows recent reports showing that IL-1β and IL-18 production can provide protection against colitis, and supported by recent GWAS studies showing that the polymorphisms which confer hypofunctional NLRP3 phenotypes are associated with the development of IBD (125). Note added in proof comes from the recent finding by Yao et al. who were using the gain of function NLRP3 R258W mice. They found that the hyperactive NLRP3 inflammasome, associated with local over-production of IL-1β, could maintain gut homeostasis resulting in strong resistance to experimental colitis. It appears that remodeled gut microbiota and increased induction of regulatory T cells were main mechanisms responsible for observed resistance (126). Therefore, it seems that a defective NLRP3 inflammasome signaling in the gut contributes to IBD, causing leaky gut and the induction of harmful immune responses against invading commensals (127).

**Figure 2:**
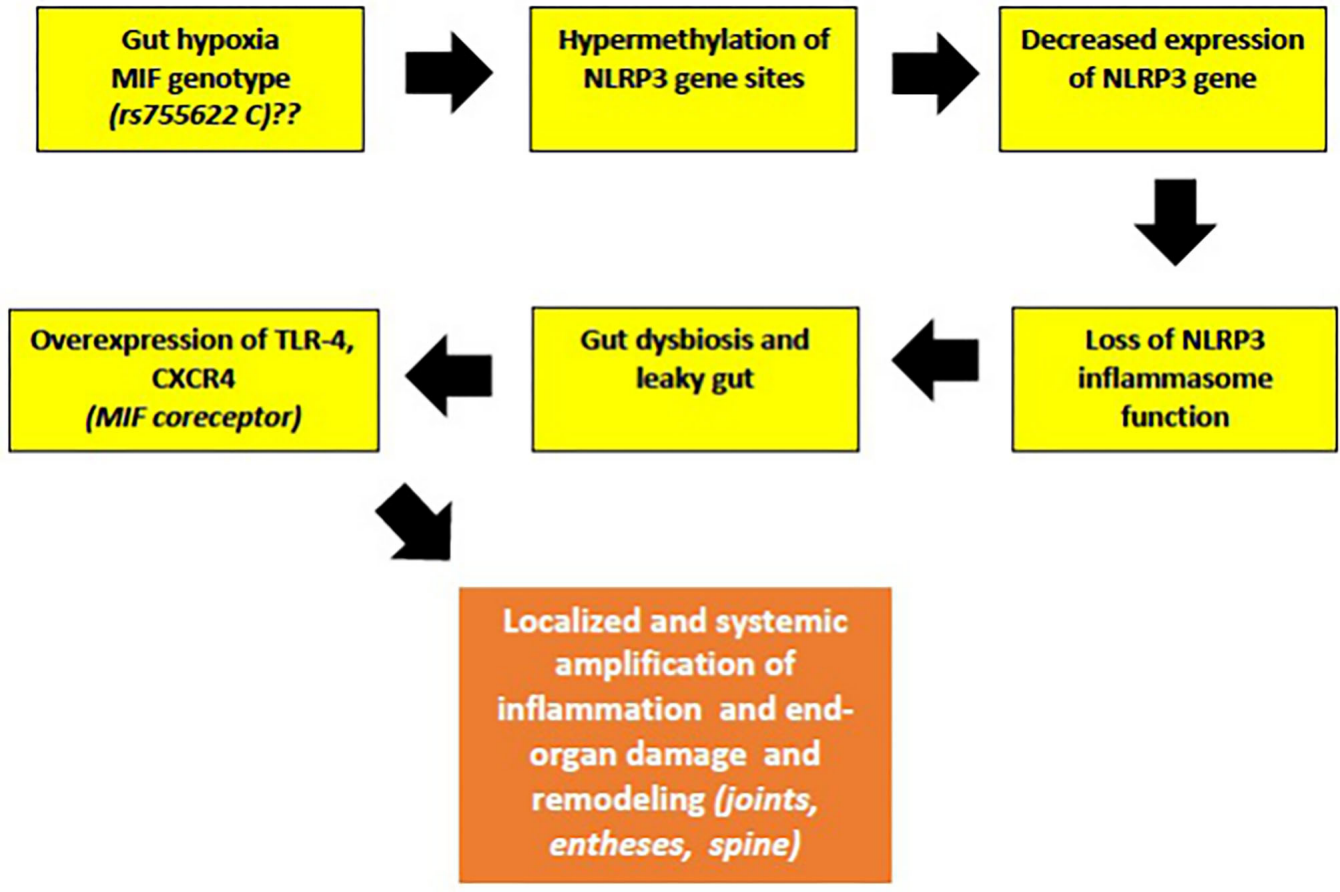
The malfunction of NLRP3 inflammasome, a key tissue damage sensor, plays an important role in various autoinflammatory and autoimmune diseases. Proposed hypothesis of decreased NLRP3 gene expression and possible role of MIF in the early phases of jSpA development (see text for description).

I further speculate that hypermethylation of NLRP3 can be promoted by certain MIF genotypes, based on similar association of MIF rs755622C allele with hypermethylation of tumor suppressors p14^ARF^ and p16^INK4a^, both encoded by Cyclin Dependent Kinase Inhibitor 2A (CDKN2A). Hypermethylation of both p14^ARF^ and p16^INK4a^ was found in normal colonic mucosal tissues of patients with UC, as well in the precancerous lesions, suggesting that UC patients with this particular inflammatory genotype of MIF may be at a higher risk for developing colonic cancer (128). This rs755622C genotype association was also observed in patients with IBD and in Chinese patients with psoriasis, but not in Turkish patients with AS (97, 98, 129). Despite that, the authors have suggested that the time of onset and the duration of AS still might be affected by rs755622C allele (129). However, this hypothesis still needs to be proven in the laboratory, along with testing for *MIF-*rs755622C allele in more patients with early-onset jSpA. In summary, the downregulated NLRP3 gene in patients with early jSpA/ErA might reflect the occurrence of a subclinical inflammation of the gut mucosa *(“low-grade IBD”*), leading to a leaky gut (Figure 2).

While the role of MIF in the early phases of SpA development is still speculative, its role in the late phases of disease is well established. Earlier reports have shown that inflammatory markers and serum MIF levels were significantly higher, and anti-inflammatory IL-10 levels were significantly lower, in patients with AS when compared to control patients. There is also a significant correlation between disease activity indices (BASFI) and MIF levels in these patients (130). It was therefore suggested that MIF may be involved in the pathogenesis of the chronic inflammation in AS. This was confirmed in a recent study where MIF was shown, not only to trigger inflammation, but also promote osteoblastic activity, suggesting its novel pathogenic role in new bone formation (NBF) in patients with SpA. It is important to mention that in SpA, NBF contributes to the disease burden independently of the pain and stiffness induced by chronic inflammation. In patients with AS increased levels of MIF have been demonstrated in the synovial fluid and ileum with a high number of MIF-producing macrophages and Paneth cells. Furthermore, increased MIF-induced TNF-α production was detected in monocytes and activated β-catenin in osteoblasts, both processes involved in promotion of the mineralization of osteoblasts leading to NBF causing spinal progression (131).

The level of expression of microRNA-451 was recently found to be lower in PMBCs of patients with AS, while MIF expression in PMBCs was significantly increased compared with those with pSpA and controls, indicating that MiR-451 suppresses inflammatory response in patients with AS by targeting MIF (132). Similarly to AS, the anti-inflammatory and anti-migratory effects of miR-451 that resulted in suppression of MIF, IL-6, TNF-α or RANTES expression, have been described *in vitro* in dendritic cells and synovial fibroblasts of RA patients and *in vivo* in mice with collagen-induced arthritis (133, 134).

In the joints themselves, MIF is also involved in synovial angiogenesis and neovascularization enhanced by loss of autophagy/ mitophagy (135). A dysregulation of these mechanisms is a critical mechanisms in the progression of inflammatory arthritis, including SpA (136). Transgenic mice overexpressing MIF exhibit high-turnover osteoporosis, while in different animal models MIF is able to enhance osteoclastogenesis through downmodulation of SDF-1 production in bone tissue and chemoattraction of circulating CXCR4+ osteoclast precursor cells (OCPs) (137–139). Furthermore, MIF (-/-) and CD74(-/-) mice also exhibit a practical absence of osteoclasts at the synovium-bone junction, as well as reduced osteoclast-related gene expression. This indicates that MIF and CD74 accelerate RANKL-induced osteoclastogenesis, suggesting that MIF contributes directly to inflammation and bone erosion in those animals (139). Nevertheless, the variety of bone pathology seen in SpA is unique in medicine and includes increased bone turnover, bone loss, osteitis, osteolysis and erosion, osteoproliferation as well as NBF, either at peripheral (enthesophytes) or axial (syndesmophytes) skeletal ligament, or tendon entheses and osteosclerosis (140). Notably, these effects can be present concurrently in the same patient.

**Figure 3:**
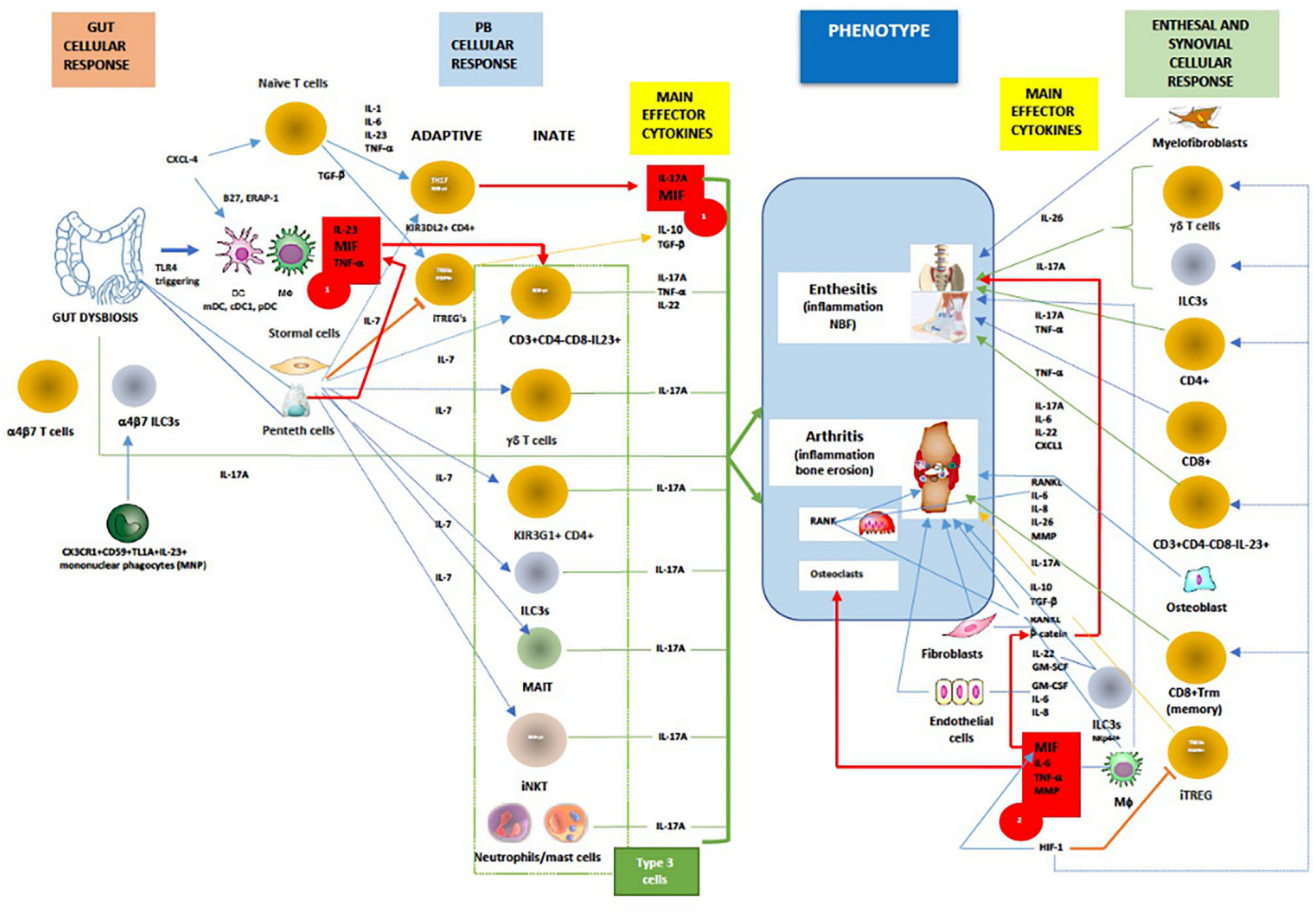
In general, MIF is an proximal mediator of host defense that up-regulates the expression of pattern recognition receptors (eg TLR-4) and perpetuates inflammatory responses by inhibiting activation-induced apoptosis in monocytes and macrophages. As mentioned above in chronic spondyloarthritis the inhibitory effect of glucocorticoids on cytokine mRNA translation may be counter-regulated by MIF. Furthermore, dysbiosis could trigger TLR-4 ligands to induce ileal production of MIF, especially in CD163+ macrophages, DCs and Paneth cells, which consequently activates CD14+ monocytes to produce TNF-α/IL-23 and primes adaptive and innate (Type 3) immune cells to produce IL-17A (*Red box 1*). Chemokine CXCL4 (*MIF co-receptor*) promotes IL-17 production in human CD4^+^ T cells by acting both directly on CD3/CD28-activated human (naive) CD4^+^ T cells or indirectly via myeloid antigen presenting cells (mDCs). On the other hand, IL-7 is a cytokine that, better than IL-23, stimulates IL-17 production in both innate and adaptive immunity, and suppress the function of regulatory T cells (Treg). High levels of IL-7 have been confirmed both in the intestinal tissue and in the inflamed synovium of patients with AS. The tissue-resident γδ T cells, ILC3s, circulating and/or gut-derived α4β7 γδ T cells, T_H_17 cells, or mucosal associated invariant T (MAIT) cells all are known to promote IL-17-driven joint/entheseal inflammation. MIF, along with TNF-α and other pro-inflammatory cytokines, also induces synovitis and enthesitis with bone erosions. Joint hypoxia induces hypoxia inducible factor (HIF) expression that augments inflammation-promoting Th17 cell development through recruitment to the IL-17 promoter. In parallel, HIF-1, by binding to Foxp3, restricts regulatory T cell (Treg) development. Through positive feedback loop HIF-1 induces MIF, which in turn causes HIF-1 expression via the MIF receptor (CD74). In contrast to early phases of SpA, axial inflammation is not dependent on IL-23, but rather on IL-17A and most likely MIF. MIF is inducing mineralization of primary osteoblasts in a dose-dependent manner, upregulates genes involved in osteogenesis and triggers stabilization of a known mediators of osteoblastic activity, namely β-catenin and wingless protein ligand (Wnt). (*Red box 2*). Beside MIF, upregulated PGE2, IL-1β, TNF-α, IL-17A, IL-22, IL-26 and IL-23, BMP-2, calcium-sensing receptor CaSR-PLCγ-**signaling** and downregulated sclerostin, ankylosis progressive homolog (ANKH) and Dikkopf-1 (DKK-1) protein are important pathways involved in bone remodeling and tissue repair. Finally, activation of both the canonical Wnt/β-catenin and noncanonical Wnt/PKCδ pathways is required for inflammation-induced new bone formation (NBF) in SpA. Taken all together, it still unknow what is/are the most important factors in NBF, even TREGS were recently proposed to paradoxically, via IL-10, promote NBF through suppressing Th17 production [adapted modified from (80, 84, 87, 119, 130–175)]. Red line/box- MIF pathway; green line -IL-17A production; orange line- iTREG suppression and signaling; dotted blue line-IL-7 signaling; blue line-cellular stimulation by other cytokines.

The immunopathogenesis of SpA, with the complex interactions of cellular and main effector cytokine network mediated by MIF, are displayed in Figure 3.

## Putting It All Together

Similar to adults, juvenile spondyloarthritis consists of chronic inflammation, articular bone erosions and pathologic new bone formation. Based on these differences with prototypical autoimmune diseases, such as rheumatoid arthritis or other connective tissue diseases, SpA may be better classified among autoinflammatory diseases (176). Children with clavicular cortical hyperostosis (CCH), a rare manifestation of jSpA, show complex patterns of gene expression related to several inflammatory pathways. These include STAT3 downregulation, B-cell activation, apoptosis, and MAP kinase with upregulated TRPM3/7 Ca^++^ channels, and the most interestingly, genes closely linked to autoinflammatory diseases PTPN12 and MEFV. Interestingly, stimulation of TRPM3/7 Ca^++^ channels can provide a second signal for NLRP3 inflammasome activation suggesting that CCH might be indeed an early autoinflammatory presentation of jSpA [manuscript in preparation, (177)].

**Figure 4:**
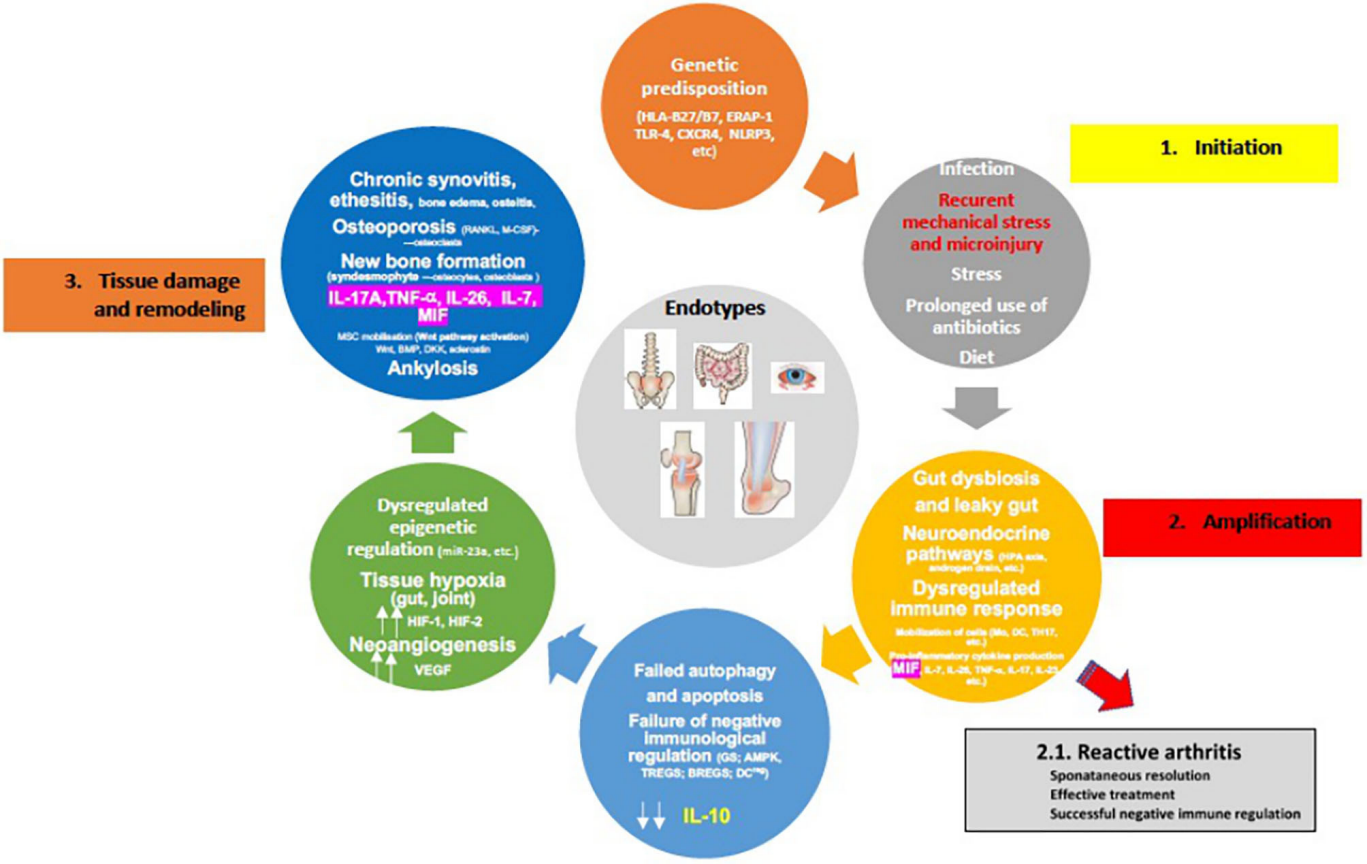
Proposed pathophysiology of spondyloarthritis development (for details please see text). [Adopted modified from (154)].

A crucial event in the early stages of SpA appears to be the strong association of osteitis with low-grade IBD, confirmed in children with ErA by elevated concentration of fecal calprotectin (fCAL), a surrogate marker of gut inflammation (178, 179). Additionally, early studies with colonoscopy have shown that patients with SpA who had sub-clinical inflammation were more likely to have active arthritis on follow up, in particular in the hip, emphasizing therefore the prognostic value of this finding (180, 181). Nonetheless, the cell types that are principally involved in local inflammation in human SpA remain largely unclear (Figure 3). Circulation of immunological cells from the intestines (e.g., entero-synovial circulation) to the entheses, synovium and spine permits the enthesitis and synovitis to become chronic. The separation between the innate and adaptive immune system is largely artificial as neither works in isolation and cross-talks are well reported. The SpA also requires specific innate and adaptive immunological events targeting the synovium with several processes that run in parallel, such as dysregulated epigenetic control, tissue hypoxia (gut and joint) and neoangiogenesis, all leading to the final stage of tissue damage and remodeling characterized by chronic synovitis and enthesitis, syndesmophyte formation and ankylosis (Figure 4).

However, considering that the frequencies of HLA-B*27 alleles and ERAP1 polymorphisms (“*first hit*”) are ethnic-specific, it is important to understand that jSpA pathogenesis could well be the result of various combinations of these mechanisms in different populations (182, 183). It is also important to underline sex differences. Examples include SLE and adult AS where different clinical phenotypes exist in males and females. Therefore, different sexes may require different biomarkers for proper diagnosis of the same disease (184). Nevertheless, two scenarios of disease development are possible: some patients who had reactive arthritis or early undifferentiated form like ErA, can reach remission (“*second hit*”), but the majority of the patients progresses to active chronic disease *(“multiple hits”*). The neuroendocrine immune response of the HPA axis and sympathetic nervous system, intended to overcome a transient inflammatory episode, are uncoupled and can therefore lead to immune cell metabolic disease in the context of erroneous energy regulation (45, 66, 175). Furthermore, failed autophagy and apoptosis of immune cells, in addition to failure of negative immune regulation (e.g., immune suppression) due to decreased GC production (*high MIF production*), blocked AMP-activated protein kinase (AMPK) pathway, and decreased IL-10 production by TREGs, BREGs, regulatory DCs most likely due to IL-7/HIF-1 production, collectively result in the progression to chronic inflammation and subtype/endotype differentiation (185, 186) (Figure 4). Of note is that the activated AMPK, and mammalian target of rapamycin (mTOR), a downstream molecule of activated AMPK, represent key control points of a series of inter-connected inflammatory signaling pathways. These include NF-κβ and JAK/STAT, crucial drivers of maintaining energy balance, cytokine signaling, cell growth, and apoptosis (187). Interestingly, in the HLA-B27/hβ2 transgenic rat model where *in vivo*, prophylactic treatment of rats with rapamycin (m-TOR inhibitor) significantly inhibited the development and severity of inflammation in peripheral joints and spine (*arthritis and spondylitis*), with histological evidence of reduced bone erosions and new bone formation, all hallmarks of SpA (188). This is relevant in view of the fact that mTOR pathway has been indeed activated in SpA synovitis, and because mTOR blockade by rapamycin or metformin in mouse model stops osteoclastogenesis. In humans with AS, that blockade also attenuates inflammation, inhibits production of IL-17A and TNF-α, bone remodeling and new periosteal bone formation (189–192). Also, rapamycin *in vitro*, may reduce inflammation in SpA by promoting autophagy of misfolded HLA-B27 (193).

In undetermined disease stage of jSpA, without well-defined and serological testing, genomic and/or imaging biomarkers become crucial because, despite biologic therapy, fewer than half of children achieve reach long-term and sustainable remission off medication 5 years after diagnosis (2, 194). Similarly, treatment of the various bone pathology in SpA remains an unmet clinical need. Although the beneficial effect of anti-TNF-α therapy might not only neutralize the effects of TNF-α, but also down-regulate Th17 and Th17-related cytokines associated with up-regulating the TREG/TGF-β axis in responders, this can also passively cause new bone formation since TNF-α stimulates the expression of DKK-1. DKK-1 in turn suppresses signaling by Wnt, promoting consequently osteoblast and osteoclast formation as well as differentiation induced by BMP-2 (195).

The definition of disease subtypes on the basis of underlying pathophysiology and the concept of endotypes has emerged more recently. Phenotypes/endotypes are dynamic, clearly overlapping and may evolve into one another, thus making clear-cut definitions somehow difficult. Nevertheless, a phenotype-/endotype-based classification approach could direct toward the application of personalized/precision medicine in the SpA field. Discoveries from basic science research might, as mentioned above, define multiple complex molecular pathways involved in the pathogenesis of jSpA, which may provide biomarkers for the molecular endotyping of this complex disease. In addition, these molecular pathways might reveal potential therapeutic targets. An endotype might consist of several complex mechanisms that cannot be clearly separated into “*pure single molecular mechanism*” thus being a “complex” endotype (196). Therefore, new powerful biomarker like fCAL that is able to differentiate various JIA subtypes, would allow us to precisely define various potential endotypes of jSpA. Down that line it was recently demonstrated that in patients with AS, a small RNA molecule, miR-199a-5p was downregulated in T cells and associated with radiographic severity of disease when compared to controls (197). MiRNA-199a-5p expression levels also showed significant negative correlations with the Ankylosing Spondylitis Disease Activity Score (ASDAS) and modified Stoke Ankylosing Spon dylitis Spinal Score (mSASSS) of AS patients. It turns out that, in T cells of AS patients, miR-199a-5p has a novel role in regulating autophagy by modulating the mTOR signaling though direct inhibition of Rheb. Rheb is known to inhibit T cells autophagy and promotes pro-inflammatory cytokine production by activating mTOR signaling (197). These data suggest that miR-199a-5p participates in the regulation of AS pathogenesis by affecting T cell autophagy and mTOR inhibition (197). In addition, the level of expression of another miR-451 was lower in AS PBMCs than in both pSpA and control PBMCs, but MIF expression was significantly increased in AS PBMCs compared to AS patients and with greater radiographic damage. It turns out, that overexpression of miR-451 suppresses the MIF (132). These findings suggest miR-451/MIF may be a novel therapeutic target in the treatment of SpA. Besides that, epigenetics could potentially be used as preventive, diagnostic, and therapeutic biomarkers and should be included in any future jSpA classification and determination of endotypes.

Well established treatment for jSpA still includes NSAIDs, but only sulfasalazine, as one of the conventional DMARDs, was found to be effective in a randomized double blind placebo controlled trial in 33 patients with jSpA after 26 weeks treatments (198). While adult with SpAs respond well to treatments that include TNF-α or IL-17-targeting biologics, they are mostly unresponsive to abatacept or MTX treatment (80, 199). Secukinumab clinical trial in children with jSpA has recently been completed (ClinicalTrials.gov: NCT03031782) but among other IL17 blocking agents, such as ixekizumab and brodalumab, that were proven to effective for adult axSpA and psoriatic arthritis, only clinical trial of ixekizumab is apparently planned in jSpA (ClinicalTrials.gov: NCT04527380) (200). Also, clinical trials of JAK inhibitors are underway in patients with JIA, including patients with ERA and psoriatic arthritis (ClinicalTrials.gov: NCT02592434, ClinicalTrials.gov: NCT03773978) (200). JAK inhibitors, in particular Tofacitinib, has shown similar efficacy to TNF inhibitors in adult SpA, including axSpA and psoriatic arthritis (201, 202).

Finally, to ultimately improve treatment efficacy and long-term outcome of patients with jSpA, consideration should be given for the use of new drugs such as iguratimod (IGU) that target simultaneously MIF, IL-17A and TNF-α, or for those that only target IL-7, m-TOR, IL-26 and/or ERAP 1 (203, 204). Blockage of MIF by a monoclonal antibody provides *in vivo* antirheumatic effects, suggesting MIF as a suitable target for antirheumatic therapy (205). Furthermore, in recent experiments, treatment of RA patients with histone deacetylase inhibitors (HDACi) downregulate MIF, in particular with two distinct orally active molecules MS-275 and SAHA. They have shown *in vivo* anti-inflammatory activities in preclinical models of rheumatoid arthritis, and both MS-275 and SAHA strongly suppress MIF protein expression by interfering with the MIF transcriptional machinery in RA synovial fibroblasts (206). Givinostat, a pan-class I/II HDACi, is currently being investigated in JIA but no data about its potential use in jSpA is currently available (207). An additional benefit of anti-MIF therapy is that it could in addition be steroid-sparing in patients with chronic steroid dependence or refractory rheumatic disease requiring daily steroid therapy. In children and adults, combination of two biologic agents is not well documented. Safety of rituximab in combination with other biologic agents (*adalimumab, etanercept, infliximab*) in adults with RA was reported as an open-label study (208). Rigby et al. showed that no serious adverse events occurred within 24 h of any rituximab infusion, and that efficacy improved at week 48 compared with that at week 24 (208). However, none of the biologic combination therapy have ever been studied in children, but favorable adverse reaction profiles, with not significant increase in infection rates with mono biologic therapy, might stimulate future researchers to consider combination therapy in children as well. Finally, another interesting approach was performed using bispecific antibody where combining a well-established anti-TNF therapeutic domain [single-chain variable fragment (scFv) of adalimumab with a synovial tissue specific targeting domain (scFv-A7) (e.g., scFv-A7 antibody) was located on the human arthritic synovium *in vitro* and in a synovium xenograft in severe combined immune deficient (SCID) mouse model (209). This study provided the first description of a BsAb capable of direct drug delivery to synovium with potential applications to other existing biologics. In practical terms, due to the improved potency, the use of such BsAb molecules in the clinical care of chronic arthritis like jSpA may offer reduced duration of treatment and consequently reducing the associated healthcare costs.

Finally, future network analysis using multiomics approach to integrate emerging forms of data from multiple platforms, has the potential to further highlights overall imunopathogenesis of the jSpA and offer true biological classification of childhood arthritis as suggested recently (210, 211).

## Author Contributions

The author confirms being the sole contributor of this work and has approved it for publication.

## Conflict of Interest

The author declares that the research was conducted in the absence of any commercial or financial relationships that could be construed as a potential conflict of interest.

## Publisher's Note

All claims expressed in this article are solely those of the authors and do not necessarily represent those of their affiliated organizations, or those of the publisher, the editors and the reviewers. Any product that may be evaluated in this article, or claim that may be made by its manufacturer, is not guaranteed or endorsed by the publisher.
